# Decompression *via* unilateral biportal endoscopy for severe degenerative lumbar spinal stenosis: A comparative study with decompression *via* open discectomy

**DOI:** 10.3389/fneur.2023.1132698

**Published:** 2023-02-22

**Authors:** Bing Tan, Qi-Yuan Yang, Bin Fan, Chuang Xiong

**Affiliations:** ^1^Department of Spine Surgery, The Third Hospital of Mianyang, Sichuan Mental Health Center, Mianyang, China; ^2^Department of Orthopedic, The First Affiliated Hospital of Chongqing Medical University, Chongqing, China

**Keywords:** unilateral biportal endoscopy, open discectomy, unilateral laminectomy bilateral decompression, severe degenerative stenosis, elderly

## Abstract

**Background:**

Previous studies have shown that the Unilateral Biportal Endoscopy is an effective and safety surgery for sufficient decompression of degenerative lumbar spinal stenosis. However, data are lacking in terms of its benefits when compared with conventional open lumbar discectomy (OLD), especially in patients with severe degenerative lumbar spinal stenosis (DLSS).

**Aim:**

To compare the clini cal outcomes of two types decompressive surgery: unilateral biportal endoscopy-unilateral laminectomy bilateral decompression (UBE-ULBD) and conventional open lumbar discectomy (OLD) in severe degenerative lumbar spinal stenosis (DLSS).

**Methods:**

We retrospectively analyzed patients who underwent UBE-ULBD (*n* = 50, operated at 50 levels; UBE-ULBD group) and conventional open lumbar discectomy (*n* = 59, operated at 47 levels; OLD group) between February 2019 and July 2021. All patients were diagnosed with severe stenosis based on the Schizas classification (Grade C or D) on MRI. We compared radiographic and clinical outcome scores [including the visual analog scale (VAS), Oswestry Disability Index (ODI), and Zurich Claudication Questionnaire (ZCQ)] between the 2 groups at 1 year of follow-up. The radiographic evaluation included the cross-sectional area (CSA) of the thecal sac and paraspinal muscles on MRI. Fasting blood was drawn before and 1 and 7 days after the operation to detect creatine kinase (CK). Surgical data perioperative complications were also investigated.

**Results:**

The baseline demographic data of the 2 groups were comparable, including VAS, ODI and ZCQ scores, the cross-sectional area of the thecal sac and paraspinal muscles and creatine kinase levels. The dural sac CSA significantly increased post -operatively in both groups, which confirmed they benefited from comparable decompressive effects. The operative duration in the OLD group was less than the UBE-ULBD group (43.9 ± 5.6 min vs. 74.2 ± 9.3 min, *p* < 0.05). The OLD group was associated with more estimated blood loss than the UBE-ULBD group (111.2 ± 25.0 ml vs. 41.5 ± 22.2 ml, *P* < 0.05). The length of hospital stay (HS) was significantly longer in the OLD group than in the UBE-ULBD group (6.8 ± 1.6 vs. 4.0 ± 1.4 days, *P* < 0.05). The VAS, ODI, and ZCQ scores improved in both groups after the operation. Serum creatine kinase values in the UBE-ULBD group were significantly lower than in the OLD group at 1 day after surgery (108. 1 ± 11.9 vs. 347.0 ± 19.5 U/L, *P* < 0.05). The degree of paraspinal muscle atrophy in the UBE-ULBD group was significantly lower than in the OLD group at 1 year (4.50 ± 0.60 vs. 11.42 ± 0.87, *P* < 0.05).

**Conclusions:**

UBE-ULBD and conventional OLD demonstrate comparable short-term clinical outcomes in treating severe DLSS. However, UBE-ULBD surgery was associated with a shorter hospital stay, less EBL and paravertebral muscle injury than OLD surgery.

## Introduction

Degenerative lumbar spinal stenosis (DLSS) is one of the most common spine diseases in the elderly population, affecting approximately 103 million people worldwide (1). It is widely acknowledged that DLSS can result in symptomatic compression of the neural elements, and early surgical treatment is recommended if conservative treatment is ineffective (2).

Large-scale decompression of traditional open laminectomy may lead to lumbar instability, resulting in persistent low back pain after operation (3, 4). Although lumbar fusion and internal fixation after decompression maintain the stability of the decompression segment, it increases the biomechanical load of the adjacent segments of the spine and sacroiliac joint, accelerates the degeneration of the adjacent segments, and also may result in back hip and lower limb pain (4, 5). In addition, this operation yields more significant trauma, intraoperative bleeding, complications and costs.

Lumbar interlaminar fenestration decompression yields less damage to the bone structure of the spine and the surrounding soft tissue than traditional open laminectomy but can damage the paravertebral muscle and its dominant nerve (6), leading to early paravertebral muscle edema, late muscle denervation, fat degeneration and atrophy, and postoperative low back pain. In recent years, UBE has been widely used in DLSS, bringing advantages such as flexibility, convenience, less tissue damage under double-channel endoscopic separation and a good clinical curative effect (7, 8).

Direct posterior bilateral decompression is indicated for severe DLSS. However, the learning curve of percutaneous endoscopic transforaminal discectomy (PETD) is steep, and the operating equipment is limited by the rigid sleeve, which accounts for the difficulty and high risks associated with bilateral decompression (9). With the development of spinal endoscopy technology, UBE can be used to treat DLSS. A previous study showed that UBE-ULBD is an effective and safe surgery for sufficient decompression of DLSS with satisfactory early follow-up outcomes (10). Nevertheless, no comparative study has hitherto assessed the clinical efficacy of UBE-ULBD and OLD in treating single-segment severe DLSS. This study aimed to investigate the clinical outcome of UBE-ULBD and compare it with conventional OLD for severe DLSS diagnosed on preoperative MRI.

## Materials and methods

### General information

This study was approved by the Committee of Medical Ethics and the Institutional Review Boards of our hospital (2022, Reviewed, No. 13).We retrospectively reviewed the clinical data of patients who underwent UBE-ULBD and OLD in a single academic institution between February 2019 and July 2021. Written informed consent was waived due to the retrospective nature of the study. Data for OLD were derived from 2019 to 2021, whereas data for UBE-ULBD were from 2020 to 2021 when the UBE-ULBD technique was used for severe DLSS. The inclusion criteria were: (1) aged >18 years; (2) Single level DLSS; (3) minimum of 1 year of follow-up; (4) Schizas Grade C or D (severe central stenosis) on preoperative MRI [Grade A, cerebrospinal fluid (CSF) is clearly visible inside the dural sac; Grade B, rootlets occupy the entire dural sac but can still be individualized; Grade C, rootlets cannot be individualized with posterior epidural fat and invisible CSF; Grade D, rootlets cannot be individualized without posterior epidural fat]; (5) All patients had symptoms associated with lower limb neurological impairment with failure to respond to conservative treatment for at least 3 month. Patients were excluded for the following reasons: (1) Previous history of lumbar surgery; (2) Presence of lumbar spondylolisthesis, lumbar instability, or degenerative deformity with Cobb angle >20°;(3) Spinal stenosis caused by lumbar tumors, tuberculosis or fractures; (4) Severe illness affecting anesthesia or surgery; (5) The follow-up period was shorter than 1 year. Consequently, this study included 51 patients (operated at 51 vertebral levels; UBE-ULBD group) who underwent unilateral biportal endoscopy unilateral laminectomy bilateral decompression (UBE-ULBD) and 59 patients (operated at 59 vertebral levels; OLN group) who underwent conventional open lumbar discectomy (OLD).All surgeries were performed by a single spinal surgeon.

### Surgical techniques

#### UBE-ULBD

**Figure 1:**
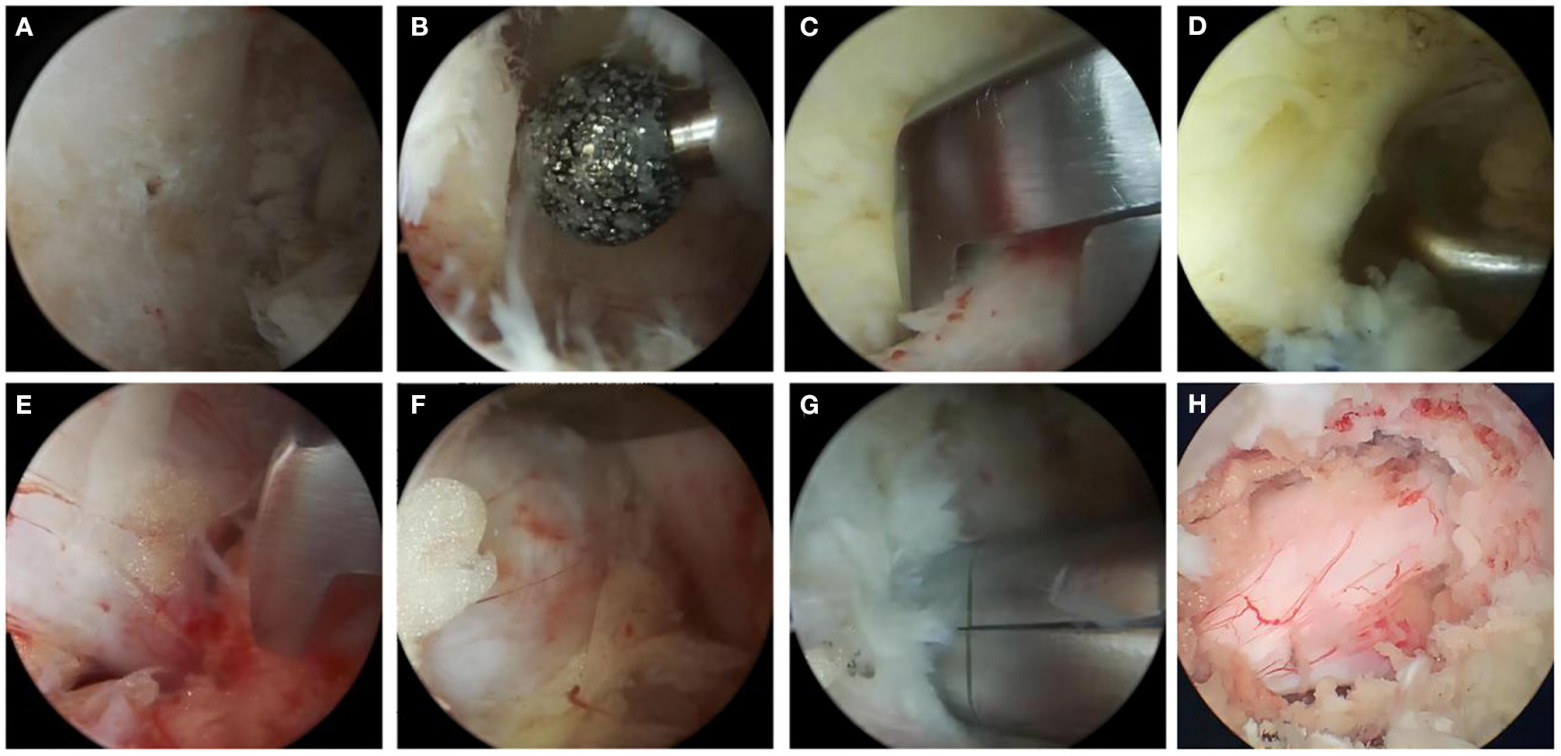
The operation process of UBE-ULBD technique: **(A)** Plasma cutter separates soft tissue on the surface of bony lamina and ligamentum flavum to establish endoscopic workspace. **(B–D)** Using pliers bite or grinding drill to remove the upper and lower edge of the lamina bone, exposing the starting and ending points of the ligamentum flavum. **(E)** The gun pliers completely bite off the ligamentum flavum. **(F, G)** exposing and removing intervertebral disc, plasma knife head for annulus fibrosus formation. **(H)** Relaxed dural and nerve roots after full decompression. One typical cases with Single level severe DLSS received UBE-ULBD.

The lesioned intervertebral space was located by fluoroscopy, and the side with severe symptoms was the operative side.The syringe needle was oriented directly opposite to the lower edge of the lamina and the junction area of the spinous process root as observed on lateral fluoroscopic view. In the AP view, the needle was 1 cm lateral to the spinous process on the operative side. Markings were made 1 cm above or below this point. After transverse incisions were created for the portals, serial dilators were inserted followed by transparent cannulas over the dilators. Water influx was then connected to the endoscopic portal inserted *via* the viewing cannula. A radiofrequency probe was used to clean the soft tissue and stop bleeding, and the intervertebral space was exposed. A guiding rod was inserted and positioned under fluoroscopy. In the AP view, the endoscopic tube and the guiding rod intersected at the intervertebral space, and the guide rod was anchored at the lower edge of the upper vertebral lamina.Bilateral partial laminectomy and medial facetectomy were performed. The nerve root canal entrance and lateral recess were carefully expanded to achieve decompression. Then, decompression was performed across the dorsal side of the dural sac, and the herniated disc was simultaneously resected (Figure 1). After adequate hemostasis, the equipment was withdrawn, drainage tubes were placed, and the incision was closed.

#### OLN

A posterior median incision was made over the spinous process under fluoroscopy, paravertebral muscles were dissected and the upper and lower edge of the adjacent lamina and the medial part of the articular process were removed for fenestration, while the lateral semi articular process was removed. The thickened ligamentum flavum and compressed tissue of dura mater and nerve root were removed to fully decompress the dura mater and nerve root. Contralateral decompression was conducted using the same approach and the contralateral recess decompression was performed until the contralateral nerve roots were decompressed, when required. After achieving adequate hemostasis, a drainage tube was placed and the incision was closed.

### Demographic and perioperative data collection

**Figure 2:**
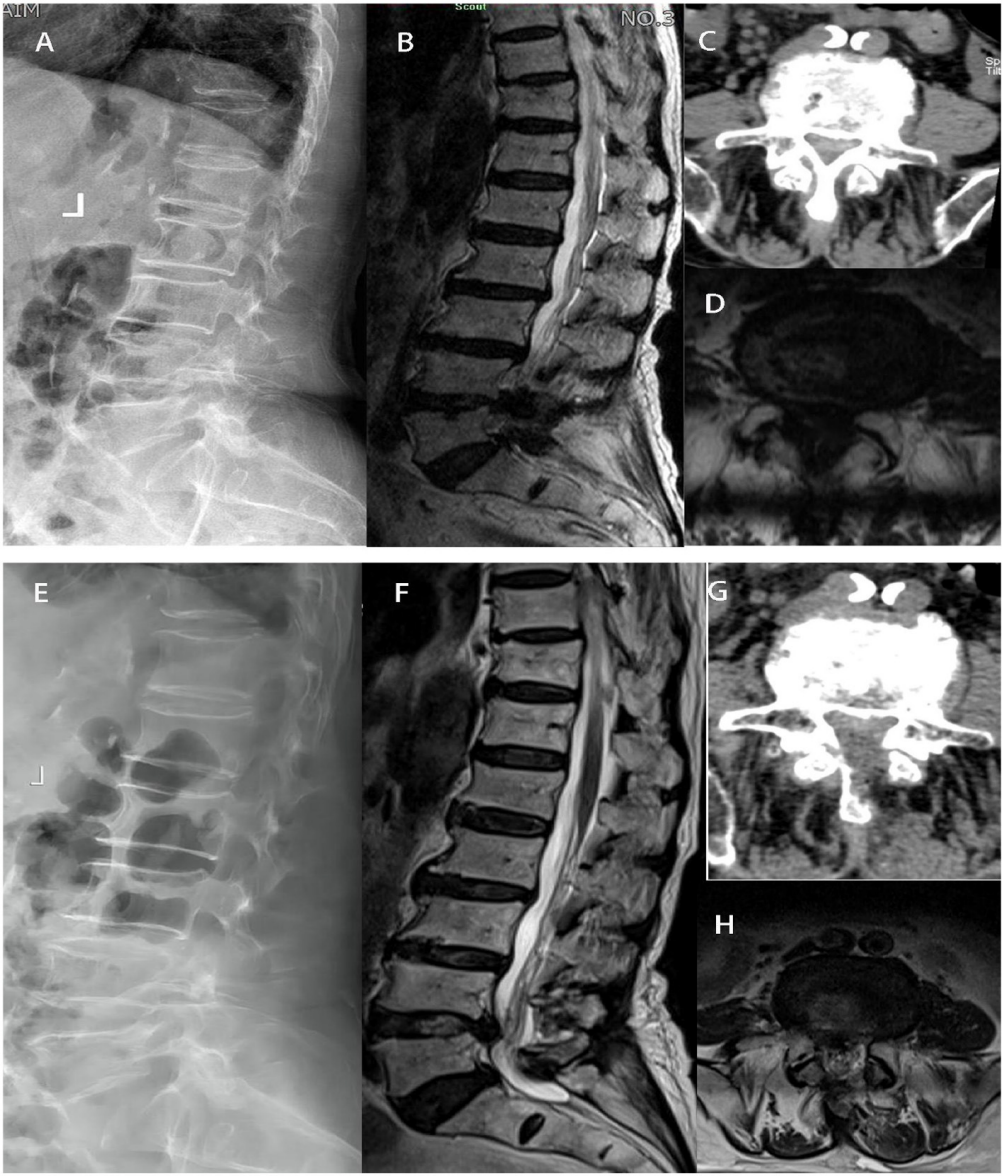
In group UBE-ULBD, a 88-year-old woman suffered from low back pain accompanied with intermittent claudication more than 6 years. Preoperative Xray **(A)**, MRI **(B, D)**, and CT **(C)** examinations showed severe DLSS (Schizas Grade D) at L4/L5 level. The patient received UBE-ULBD and symptoms significantly relieved after the surgery. Postoperative X ray **(E)**, Post-operative CT/MRI indicated completed decompression was achieved at L4/L5 **(F–H)**.

Patient demographic and perioperative data were reviewed based on medical records. Demographic data included age, gender, body mass index (BMI), smoking status, symptom duration, length of stay, follow-up period, and preoperative diagnosis. Perioperative variables included the operative level, operative time, estimated blood loss and intra- and postoperative complications (within 1 year postoperatively). Creatine kinase was recorded before and 1 and 7 days after the operation. Typical diseases are shown in Figure 2.

### Clinical and radiographic outcomes

Clinical outcome was evaluated using the visual analog scale (VAS), Oswestry Disability Index (ODI) and Zurich Claudication Questionnaire (ZCQ) for DLSS, which includes subjective symptoms and objective clinical signs. The VAS, ODI and ZCQ scores were calculated preoperatively and at 1 week, 6 months, and 12 months postoperatively.

The radiographic evaluation included the cross-sectional area (CSA) of the thecal sac and paraspinal muscles based on MRI scans performed preoperatively and at 1 year of follow-up. The CSA of the thecal sac was measured at the disc level using T2-weighted axial MRI preoperatively and at the 1-year follow-up. To eliminate inter-individual heterogeneity, the improvement rate of dural sac CSA was analyzed in this study. The improvement rate of dural sac CSA was calculated according to the following formula: (postoperative dural sac area–preoperative dural sac area) / preoperative dural sac area×100%. The multifidus and erector spinae muscles were measured, including the non-muscular tissue between them, together as one muscle unit and considered the paraspinal muscles. The CSA of the paraspinal muscles was measured at the disc level using MRI, preoperatively and at the 1-year follow-up using Image J software (NIH, Bethesda, MD, USA). The ratio of muscle CSA variation (RCV) was calculated according to the following formula: Last CSA/ Preoperative CSA×100%. The degree of paravertebral muscle atrophy was calculated according to the following formula: 100%-RCV.

### Statistical analysis

All data were expressed as mean ± standard deviation (SD) unless otherwise specified. A board-certified spine surgeon blinded to the procedure assessed all radiography results. The interobserver reliability was assessed using intraclass correlations with data measured by one of the co-authors and classified as poor (0–0.39), moderate (0.4–0.74), or excellent (0.75–1). For continuous variables, within-group and between-group differences were detected using Student t-tests and paired *t*-tests, respectively. To compare categorical variables, a Chi-square analysis was performed.Statistical significance was set at *p* < 0.05. All statistical data analyses were executed using SPSS software (SPSS Inc, Chicago, IL, USA, version 23.0).

## Results

A summary of the demographics and baseline characteristics of the groups is presented in Table 1. Analysis of the baseline demographics (Table 1) showed no statistical differences between the two groups. Schizas Grade C (severe stenosis) was dominant in both groups (UBE-ULBD 72% and OLD 69.5%), while the remaining patients had more severe stenosis, classified as Grade D.

**Table 1:** Patient demographic data.

**Variables**	**UBE-ULBD (*n* = 50)**	**OLD (*n* = 59)**	***p*-value**
Age (years)	64.8 ± 13.6	66.3 ± 12.7	0.554
Sex (%)			
Female	21 (42%)	27 (45.8%)	0.693
Male	29 (58%)	32 (54.2%)	
BMI (kg/m^2^)	26.4 ± 2.9	27.3 ± 2.3	0.102
Operative level, *n* (%)			
L4-5	27 (54%)	33 (55.9%)	0.864
L5-S1	23 (46%)	26 (45.1%)	
Schizas classification, *n* (%)			
C	36 (72%)	41 (69.5%)	0.774
D	14 (28%)	18 (30.5%)	
Duration of disease (days)	35.1 ± 9.2	36.3 ± 8.2	0.498
Comorbidity			
Hypertension	37 (74%)	43 (72.9%)	0.972
Cardiopathy	29 (58%)	33 (55.9%)	
Lung disease	35 (70%)	43 (72.9%)	
Follow-up (months)	15.3 ± 2.5	16.1 ± 2.1	0.065

BMI, Body mass index; Schizas classification on MRI.

Grade A, CSF is clearly visible inside the dural sac.

Grade B, rootlets occupy the entire dural sac but can still be individualized.

Grade C, rootlets cannot be individualized with visible posterior epidural fat and effacement of CSF space.

Grade D, no recognized rootlets and posterior epidural fat.

The OLD group was associated with a shorter operative duration than the UBE-ULBD group (43.9 ± 5.6 vs. 74.2 ± 9.3 min) and more estimated blood loss (111.2 ± 25.0 vs. 41.5 ± 22.2 ml) in the UBE-ULBD group (*P* < 0.05; Table 2). The duration of hospital stay was significantly longer in the OLD group than in the UBE-ULBD group (6.8 ± 1.6 vs. 4.0 ± 1.4 days, *P* < 0.05, Table 2). No significant difference in perioperative complications was observed with dural sac tearing (*n* = 1) in the UBE-ULBD group and dural sac tearing (*n* = 2) and incision infection (*n* = 3) in the OLD group. These complications subsided within 1 month after surgery. The dural sac CSA significantly increased postoperatively in two groups, which confirmed the two groups benefited from a comparable decompressive effect (UBE-ULBD preop: 0.83 ± 0.14 vs. postop: 1.55 ± 0.075 cm^2^, OLD preop: 0.83 ± 0.13 vs. postop: 1.54 ± 0.079 cm^2^, *P* < 0.05, Table 3).

**Table 2:** Perioperative characteristics by type of procedure.

**Variables**	**UBE-ULBD (*n* = 50)**	**OLD (*n* = 59)**	***p*-value**
Operative time (min)	74.2 ± 9.3	43.9 ± 5.6	< 0.001^*^
EBL (ml)	41.5 ± 22.2	111.2 ± 25.0	< 0.001^*^
Length of hospital stay (days)	4.0 ± 1.4	6.8 ± 1.6	< 0.001^*^
Creatine Kinase (U/L)	
Preop	61.8 ± 7.5	61.1 ± 8.3	0.687
Postoperative day 1	108 1 ± 11 9	347 0 ± 19 5	< 0 001^*^
Postoperative day 7	62.1 ± 7.4	61.6 ± 8.4	0.756
Perioperative complications, *n* (%)	1	5	0.140
Dural sac tearing	1 (2%)	3 (5.1%)	
Incision infection	0	2 (3.4%)	

EBL, Estimated blood loss.

* *p* < 0.05, the difference was significant.

**Table 3:** Radiographic outcomes by type of procedure at 1-year follow-up.

**Variables**	**UBE-ULBD (*n* = 50)**	**OLD (*n* = 59)**	***p*-value**
dural sac CSA, cm^2^			
Preop	0.83 ± 0.14	0.83 ± 0.13	0.816
1 year postop	1.55 ± 0.075	1.54 ± 0.079	0.596
Improvement percentage of dural sac	91.24 ± 32.52	91.76 ± 33.77	0.936
CSA (%)			
CSA of the paravertebral muscles (PM, cm^2^)			
Preop	32.20 ± 2.63	32.59 ± 2.46	0.422
1 year postop	30.68 ± 3.01	27.95 ± 2.46	< 0.001^*^
Degree of PM atrophy (%)	4.50 ± 0.600	11.42 ± 0.870	< 0.001^*^

CSA, Cross-sectional area.

* *p* < 0.05, the difference was significant.

Serum creatine kinase (CK) significantly increased in both cohorts and peaked 1 day after surgery, although significantly lower levels were found in the UBE-ULBD group than in the OLD group (108.1 ± 11.9 vs. 347.0 ± 19.5 U/L, *P* < 0.05, Table 2). However, there were no significant differences between the two groups preoperatively and on postoperative day seven. The CSA of the paravertebral muscles in the UBE-ULBD group was significantly greater than in the OLD group at 1 year, with a significantly lower degree of atrophy of the paraspinal muscles in the UBE-ULBD group than in the OLD group (4.50 ± 0.600 vs. 11.42 ± 0.870, *P* < 0.05, Table 3).

The ODI and ZCQ score significantly improved in both groups at 1 week, 6 months and 1 year postoperatively (*p* < 0.05, Table 4). There were no significant inter-group differences in preoperative and postoperative scores at any follow-up time point, except for the significantly higher VAS and ODI scores in the UBE-ULBD group in the OLD group on postoperative day seven (VAS:3.22 ± 0.62 vs. 3.68 ± 0.88; ODI: 37.20 ± 2.25 vs. 38.93 ± 2.43, *p* < 0.05, Table 4).

**Table 4:** Comparison of postoperative VAS, ODI, and ZCQ scores.

**Scoring system**	**UBE-ULBD (*n* = 50)**	**OLD (*n* = 59)**	***p*-value**
**VAS**			
Preop (mean score)	6.82 ± 0.94	6.53 ± 0.90	0.098
Postop (1 week)	3.22 ± 0.62	3.68 ± 0.88	0.003*
Follow-up at 6 months	2.36 ± 0.60	2.44 ± 0.75	0.541
Follow-up at 1 years	1.92 ± 0.49	1.86 ± 0.63	0.612
*p*-value (pre vs. post)	0.000	0.000	
**ODI**			
Preop (mean score)	61.84 ± 3.23	62.47 ± 3.55	0.335
Postop (1 week)	37.20 ± 2.25	38.93 ± 2.43	0.000*
Follow-up at 6 months	16.04 ± 2.36	16.97 ± 3.07	0.084
Follow-up at 1 years	13.04 ± 2.05	13.66 ± 2.36	0.150
*p*-value (pre vs. post)	0.000	0.000	
**ZCQ**			
Preop (mean score)	66.26 ± 3.24	66.78 ± 3.52	0.428
Postop (1 week)	39.22 ± 4.32	40.12 ± 4.04	0.265
Follow-up at 6 months	22.52 ± 4.32	39.22 ± 4.32	0.912
Follow-up at 1 years	39.22 ± 4.32	39.22 ± 4.32	0.523
*p*-value (pre vs. post)	0.000	0.000	

VAS, Visual analog scale; ODI, Oswestry disability index; ZCQ, Zurich claudication questionnaire;^*^p < 0.05, the difference was significant.

## Discussion

Since Kambin et al. first reported lumbar degenerative disease treatment with bilateral double channel and unilateral double channel operation in 1996 (11), there has been a growing body of evidence suggesting that lumbar stenosis can be treated *via* unilateral biportal endoscopy (7, 8). Nevertheless, conventional OLD has been the standard decompression technique for decades with a proven clinical outcome. Interestingly, in the presence of severe DLSS, OLD can be used to remove the hyperplastic and abnormal tissues that oppress the dural sac and nerve root under direct vision and retain the normal anatomical structure of the spine to the greatest extent compared with other open surgery (12–14). However, the OLD approach requires extensive stripping of paraspinal muscle tissue to expose the surgical field and greater force to constantly pull the paraspinal muscles, causing the ischemic injury of paraspinal muscle and surrounding scar formation (14). Growing evidence suggests that muscle atrophy after paraspinal muscle injury accelerates spinal degeneration, resulting in decreased spine stability, postoperative pain and dysfunction (15, 16).

Nowadays, UBE decompression procedures have become popular as an alternative to OLD for treating DLSS, and UBE-assisted unilateral laminectomy with bilateral decompression can achieve bilateral decompression without contralateral soft-tissue dissection (17, 18). Consistently, our study substantiated that UBE-ULBD could achieve good short-term clinical outcomes in treating severe DLSS. By adjusting the angle of the endoscope during the operation, the UBE technique enabled clear visualization of the compressed structures that crossed the dorsal side of the dural sac into the contralateral crypt, making it easier to achieve contralateral decompression without damaging the contralateral paravertebral muscle. This technique can avoid ipsilateral paravertebral muscle damage and achieve precise decompression to preserve the facet joints and the stability of the decompression segment (8, 17).

After the operation, back muscle exercises can be carried out early to prevent paravertebral muscle atrophy.

Moreover, damage to the dorsal spinal nerve and its branches may occur during muscle dissection in OLD, resulting in postoperative paraspinal muscle atrophy.

However, the operative space established by the water medium is minimally affected by nerves and muscles in the UBE-ULBD, accounting for the low incidence of postoperative paraspinal muscle atrophy (8, 17, 18).

The intraoperative paravertebral muscle injury causes necrosis of muscle cells under the stimulation of early inflammation, resulting in elevation of creatine kinase in peripheral venous blood after operation (19). Overwhelming evidence substantiates that fat degeneration and atrophy fibrosis can occur in paravertebral muscles after lumbar spine surgery, and MRI can accurately evaluate paravertebral muscle injury and atrophy (15, 16). This study showed that creatine kinase levels and paraspinal muscle atrophy in the UBE-ULBD group were significantly lower than in the OLD group. This finding indicated that the UBE technique could effectively reduce injury to paraspinal muscles, thereby reducing the incidence of failed back surgery syndrome and adjacent segment degeneration.

Indeed, UBE technology combines the advantages of open spinal surgery and endoscopic spinal surgery. It is an innovative application of arthroscopy in spinal surgery, especially for minimally invasive treatment of DLSS (20). The technique makes up for the limitations, including a poor operative field of view and limited operating range of the channel microscope technique using an air medium.It also offsets the poor visibility and need for special surgical instruments of intervertebral foramen technology using a water medium for the working channel (7, 21).Herein, the VAS and ODI scores of the UBE-ULBD group were significantly better than the OLD group 7 days after the operation. The VAS and ODI scores of the two groups at 6 months and 1 year after operation were significantly improved, although no significant difference was found. Consistently, the ZCQ scores of the two groups at 1 week, 6 months and 1 year were significantly improved with no significant difference. Moreover, the cross-sectional area of the thecal sac was significantly improved in both groups, with no significant difference found. The estimated blood loss and hospital stay in the UBE-ULBD group were significantly less than in the OLD group, suggesting that UBE-ULBD technology yields less damage to the surrounding bone and soft tissue, faster postoperative recovery, and can yield the bilateral decompressive effect of open surgery. However, our research was a single-center retrospective observational study with a relatively small sample size, and its results require further confirmation by a prospective multicenter study.

## Conclusion

During the treatment of severe DLSS, UBE-ULBD offsets the shortcomings of transforaminal endoscopy and microscopic channel technology. Compared with open surgery, a more consistent clinical effect is achieved, with less trauma to the paraspinal muscle and faster clinical recovery, reducing paraspinal muscle atrophy and lower lumbar pain later.

## Data availability statement

The original contributions presented in the study are included in the article/supplementary material, further inquiries can be directed to the corresponding author.

## Ethics statement

The studies involving human participants were reviewed and approved by the Medical Ethics Committee of the Third Hospital of Mianyang (Sichuan Mental Health Center). The patients/participants provided their written informed consent to participate in this study. Written informed consent was obtained from the individual(s) for the publication of any potentially identifiable images or data included in this article.

## Author contributions

The study conception and design were contributed and the first draft of the manuscript was written by BT. Material preparation, data collection, and analysis were performed by Q-YY, BF, and CX. All authors read and approved the final manuscript.

## References

[1] Katz JN Zimmerman ZE Mass H Makhni MC. Diagnosis and manage -ment of lumbar spinal stenosis: a review. JAMA. (2022) 327:1688–99. 10.1001/jama.2022.5921 35503342

[2] Zhao Y Zhu Y Zhang H Wang C He S Gu G. Comparison of bilateral versus unilateral decompression incision of minimally invasive transforaminal lumbar interbody fusion in two-level degenerative lumbar diseases. Int Orthop. (2018) 42:2835–42. 10.1007/s00264-018-3974-z 29754188

[3] Wei FL Zhou CP Liu R Zhu KL Du MR Gao HR. Management for lumbar spinal stenosis: a network meta-analysis and systematic review. Int J Surg. (2021) 85:19–28. 10.1016/j.ijsu.2020.11.014 33253898

[4] Zaina F Tomkins-Lane C Carragee E Negrini S. Surgical options for lumbar spinal stenosis. Cochrane Database Syst Rev. (2016) 11:CD012421. 10.1002/14651858.CD010264.pub2 26824399 PMC6669253

[5] Farrokhi MR Yadollahikhales G Gholami M Mousavi SR Mesbahi AR Asadi-Pooya AA. Clinical outcomes of posterolateral fusion vs. Posterior lumbar interbody fusion in patients with lumbar spinal stenosis and degenerative instability. Pain Physician. (2018) 21:383–406. 10.36076/ppj.2018.4.383 30045595

[6] Pietrantonio A Trungu S Famà I Forcato S Miscusi M Raco A. Long -term clinical outcomes after bilateral laminotomy or total laminectomy for lumbar spinal stenosis: a single-institution experience. Neurosurg Focus. (2019) 46:E2. 10.3171/2019.2.FOCUS18651 31042648

[7] Aygun H Abdulshafi K. Unilateral biportal endoscopy versus tubular micro-endoscopy in management of single level degenerative lumbar canal stenosis: a prospective study. Clin Spine Surg. (2021) 34:E323–8. 10.1097/BSD.0000000000001122 33470660 PMC8225231

[8] Park MK Son SK Park WW Choi SH Jung DY Kim DH. Unilateral Biportal endoscopy for decompression of extraforaminal stenosis at the lumbosacra -l junction: surgical techniques and clinical outcomes. Neurospine. (2021) 18:871–9. 10.14245/ns.2142146.073 35000343 PMC8752693

[9] Chen H Zhang H Yang E Ling Q He E. Percutaneous bilateral endoscopic lumbar inter-body fusion: technical note and preliminary results. Biomed Res Int. (2022) 22:671–9. 10.1155/2022/2227679 35445131 PMC9015859

[10] Heo DH Lee DC Park CK. Comparative analysis of three types of minimally invasive decompressive surgery for lumbar central stenosis: biportal endoscopy, uniportal endoscopy, and microsurgery. Neurosurg Focus. (2019) 46:E9. 10.3171/2019.2.FOCUS197 31042664

[11] Kambin P. Diagnostic and therapeutic spinal arthroscopy. Neurosurg Clin N Am. (1996) 7:65–76. 10.1016/S1042-3680(18)30406-6 8835147

[12] Kang TW Park SY Oh H Lee SH Park JH Suh SW. Risk of reoperation and infection after percutaneous endoscopic lumbar discectomy and open lumbar discectomy : a nationwide population-based study. Bone Joint J. (2021) 103-B:1392–9. 10.1302/0301-620X.103B8.BJJ-2020-2541.R2 34334035

[13] Masuda S Fukasawa T Takeuchi M Fujibayashi S Otsuki B Murata K. Reoperation rates of microendoscopic discectomy compared with conventional open lumbar discectomy: a large-data -base study. Clin Orthop Relat Res. (2022) 15:76–85. 10.1097/CORR.0000000000002322 35838602 PMC9750527

[14] Li Z Zhang C Chen W Li S Yu B Zhao H. Percutaneous endoscopic transforaminal discecto -my versus conventional open lumbar discectomy for upper lumbar disc herniation: a comparative cohort study. Biomed Res Int. (2020) 18:20–22. 10.1155/2020/1852070 32190653 PMC7072112

[15] Niemeyer F Zanker A Jonas R Tao Y Galbusera F Wilke HJ. An externally validated deep learning model for the accurate segmentation of the lumbar paravertebral muscles. Eur Spine J. (2022) 31:2156–64. 10.1007/s00586-022-07320-w 35852607

[16] Crawford RJ Filli L Elliott JM Nanz D Fischer MA Marcon M. Age- and level-dependence of fatty infiltration in lumbar paravertebral muscles of healthy volunteers. AJNR Am J Neuroradiol. (2016) 37:742–8. 10.3174/ajnr.A4596 26635285 PMC7960169

[17] Soliman HM. Irrigation endoscopic decompressive laminotomy. A new endoscopic approach for spinal stenosis decompression. Spine J. (2015) 15:2282–9. 10.1016/j.spinee.2015.07.009 26165475

[18] Nomura K Yoshida M. Microendoscopic decompression surgery for lumbar spinal canal stenosis via the paramedian approach: preliminary results. Global Spine. (2012) 2:87–94. 10.1055/s-0032-1319774 24353952 PMC3864472

[19] Portella ST Escudeiro GP Mansilla R Freitas BS de Resende MAC Fernandes FC. Predictive factors for muscle injury after posterior lumbar spinal surgery. World Neurosurg. (2019) 129:e514–21. 10.1016/j.wneu.2019.05.197 31152890

[20] Chu PL Wang T Zheng JL Xu CQ Yan YJ Ma QS. Global and current research trends of unilateral biportal endoscopy /biportal endoscopic spinal surgery in the treatment of lumbar degenerative diseases: a bibliometric and visualization study. Orthop Surg. (2022) 14:635–43. 10.1111/os.13216 35293686 PMC9002063

[21] Zheng B Xu S Guo C Jin L Liu C Liu H. Efficacy and safety of unilateral biportal endoscopy versus other spine surgery: a systematic review and meta-analysis. Front Surg. (2022) 9:911–4. 10.3389/fsurg.2022.911914 35959116 PMC9357908

